# Skeletal involvement in Gaucher disease: extent of bone disease, splenic volume, and quality of life

**DOI:** 10.1590/0100-3984.2020.0014

**Published:** 2021

**Authors:** Ricardo Andrade Fernandes de Mello, Melissa Bosi Nonato Mello, Laís Bastos Pessanha, Ana Paula Alves Fonseca

**Affiliations:** 1 Department of Internal Medicine, Universidade Federal do Espírito Santo (UFES), Vitória, ES, Brazil.; 2 Centro Capixaba de Oncologia (Cecon), Vitória, ES, Brazil.

**Keywords:** Gaucher disease, Magnetic resonance imaging, Musculoskeletal system, Hematologic diseases, Doença de Gaucher, Ressonância magnética, Sistema musculoesquelético, Doenças hematológicas

## Abstract

**Objective:**

To investigate the correlations among the extent of bone involvement, splenic volume, and quality of life in patients with Gaucher disease.

**Materials and Methods:**

This was a descriptive, prospective cross-sectional study of 18 patients with Gaucher disease who underwent 3-T magnetic resonance imaging of both femurs and the lumbar spine. Semiquantitative analyses were performed on the basis of the bone marrow burden (BMB) score. We looked for linear relationships among the variables splenic volume, quality of life score, and BMB score.

**Results:**

We identified a linear relationship between the BMB scores and splenic volume. The quality of life score showed no statistically significant relationship with splenic volume or the BMB score.

**Conclusion:**

The linear relationship between the BMB score and the splenic volume indicates that the extent of bone disease is greater in individuals with splenomegaly. No correlation was found between the BMB and quality of life scores, illustrating the insidious and silent progression of Gaucher disease.

## Abstract (pt)

**Objetivo:**

Investigar a correlação entre a extensão do envolvimento ósseo, o volume esplênico e a qualidade de vida em pacientes com doença de Gaucher.

**Materiais e Métodos:**

Estudo descritivo, prospectivo e transversal de 18 pacientes com doença de Gaucher submetidos a ressonância magnética de 3-T de ambos os fêmures e da coluna lombar. Análise semiquantitativa foi feita utilizando o escore *bone marrow burden* (BMB). Correlação linear foi estudada para as variáveis volume esplênico, qualidade de vida e escore BMB.

**Resultados:**

Uma correlação linear entre os escores BMB e volume esplênico foi demonstrada. Em relação ao índice de qualidade de vida, não foi observada correlação estatisticamente significante nem com o volume esplênico e nem com o escore BMB.

**Conclusão:**

Nosso estudo demonstrou uma correlação linear entre o escore BMB e o volume esplênico, correspondendo a maior extensão de doença óssea em indivíduos com maior esplenomegalia. Nenhuma correlação foi encontrada entre o escore BMB e a qualidade de vida, indicando a natureza insidiosa e a progressão silenciosa da doença de Gaucher.

## INTRODUCTION

Gaucher disease (GD) was the first reported lysosomal storage disease and is the most common type of lipidosis. It is caused by hereditary deficiency of the lysosomal enzyme glucocerebrosidase (or beta-glucosidase), which hydrolyzes glucosylceramide (glucocerebroside) into glucose and ceramide ^(1,2)^. The accumulation of glucocerebroside in the bone marrow leads to osteopenia, lytic lesions, pathological fractures, chronic bone pain, bone crises, and infarcts^(3,4)^. The incidence of bone neoplasms has also been shown to be higher in patients with GD^(5,6)^.

There are different types of GD, and signs and symptoms of the disease vary widely, even within the same type. It can be classified into two main groups: non-neuronopathic GD (type 1) and neuronopathic GD, in which the central nervous system is involved (types 2 and 3). Skeletal involvement is seen on the radiographs of most patients with GD, who are often asymptomatic^(7)^. The severity of bone involvement and the rate of progression vary considerably in GD, although the disease is typically more aggressive in patients who present with symptoms during childhood^(8,9)^.

The progression of many bone complications can be halted or reversed by enzyme replacement therapy (ERT), although osteonecrosis, osteosclerosis, and vertebral fracture may be irreversible, which can have a significant impact on quality of life^(10,11)^. Therefore, bone disease may be the most debilitating and incapacitating aspect of type 1 GD and should be monitored routinely and early^(12-15)^. The extent of bone marrow infiltration has been shown to be one of the most important parameters for evaluating the severity of GD, and various classifications have been used^(16-18)^. Quantifying bone involvement has therefore become increasingly important in clinical management, not only to determine the eligibility of each patient but also to monitor the effects of therapy, given that ERT is currently costly^(19,20)^.

The broad spectrum of clinical manifestations in GD and its unpredictable progression underscore the need for the use of instruments that facilitate the objective prediction of the prognosis and need for therapy^(21,22)^. Quantitative and semiquantitative scoring systems serve this purpose for a number of diseases, and the bone marrow burden (BMB) score, based on magnetic resonance imaging (MRI), has proven to be a reliable tool for demonstrating a response to ERT in type 1 GD^(3,23,24)^. Because of its high sensitivity in the detection of focal and diffuse pathologies, including bone infarction, avascular necrosis, trauma, infection, and bone marrow infiltration by Gaucher cells, MRI is the method of choice for the evaluation of bone involvement in GD. To evaluate the impact that the disease has on patients, health-related quality of life questionnaires, such as the Medical Outcomes Study 36-Item Short Form Health Survey (SF-36), are commonly applied to measures of function in clinical settings, providing self-report information on how patients perceive their functioning in life^(23)^.

The purpose of this study was to investigate the correlations among the extent of bone involvement (BMB score), splenic volume, and quality of life in patients with type 1 GD. We also attempted to determine whether self-reported physical, social, and mental functioning correlate with the extent of bone disease, which may have implications for treatment planning.

## MATERIALS AND METHODS

### Patients

This was a prospective, cross-sectional study conducted at a referral center for GD between February and October of 2014. The study population comprised 18 adult patients (10 females and 8 males) with a confirmed diagnosis of GD, characterized by decreased acid beta-glucosidase activity in peripheral blood leukocytes and clinical features consistent with type 1 disease. Two of the 18 patients (11%) had undergone splenectomy. The study was approved by the local research ethics committee, and all participating patients gave written informed consent.

### MRI

All patients underwent MRI of the lumbar spine and both femurs in a 3-T MRI scanner (Achieva; Philips, Best, The Netherlands). The lumbar spine imaging consisted of sagittal turbo spin-echo (TSE) T1-weighted sequences-repetition time/echo time (TR/TE), 344/11 ms; bandwidth, 308.8 Hz; slice thickness, 4 mm; number of signals averaged, 2; matrix, 320 × 320; field of view, 27 cm^2^; and interslice gap, 1 mm-and sagittal TSE T2-weighted sequences-TR/TE, 2,927/120 ms; bandwidth, 397.7 Hz; slice thickness, 4 mm; number of signals averaged, 3; matrix, 320 × 320; field of view, 27 cm^2^; and interslice gap, 1 mm. Femoral imaging consisted of coronal TSE T1-weighted sequences-TR/TE, 621/11 ms; bandwidth, 275.6 Hz; slice thickness, 7 mm; number of signals averaged, 2; matrix, 392 × 157; field of view, 31 cm^2^; and interslice gap, 1 mm-and coronal TSE short-tau inversion-recovery sequences-TR/TE, 3,352/120 ms; inversion time, 160 ms; bandwidth, 293.2 Hz; slice thickness, 7 mm; number of signals averaged, 2; matrix, 390 × 313; field of view, 31 cm^2^; and interslice gap, 1 mm. In addition, to allow assessment of splenic volume, abdominal images were acquired in an axial TSE T2-weighted sequence-TR/TE, 1,800/90 ms; bandwidth, 320 Hz; slice thickness, 7 mm; number of signals averaged, 2; matrix, 320 × 320; field of view, 39 cm^2^; and interslice gap, 1 mm.

### Quality of life

On the day scheduled for the MRI examination, we applied the SF-36 questionnaire, which consists of a multidimensional evaluation involving 11 items, encompassing eight domains: role-physical; functional capacity; role-emotional; bodily pain; general health; vitality; social functioning; and mental health. The version of the SF-36 employed in this study also includes a question designed to provide a comparative evaluation between the current health status and that of a year ago. Higher SF-36 scores indicate better health-related quality of life.

### Splenic volume

Splenic volume (expressed in cubic centimeters) was measured by tracing the splenic outline. To do so, we selected the appropriate regions of interest manually, respecting the contours of the spleen while excluding large vessels and fissures, from the abdominal T2-weighted TSE images obtained in the axial plane. We used OsiriX open-source software (OsiriX Foundation, Geneva, Switzerland) to trace the outline and calculate the volume.

### BMB score

Semiquantitative analysis were performed using BMB scores, in accordance with previously published criteria^(3)^, based on the evaluation of changes in bone marrow signal intensity and the extent of involvement, in the femurs and in the lumbar spine (Tables 1 and 2, respectively). Scores for the lumbar spine and for the femurs ranged from 0 to 8, resulting in a maximum overall BMB score of 16, higher scores being indicative of greater bone marrow involvement. To analyze the separate contributions of the axial and peripheral bone marrow, we subdivided the BMB scores into lumbar spine BMB (L-BMB) and femoral BMB (F-BMB) scores.

**Table 1:** F-BMB score, based on MRI signal intensity, together with the sites of femoral involvement.

Variable	Signal intensity*	F-BMB score
MRI sequence		
T2-weighted	Hyperintense	2
T2-weighted	Slightly hyperintense	1
T2-weighted	Isointense	0
T2-weighted	Slightly hypointense	1
T2-weighted	Hypointense	2
T2-weighted	Mixed type	3
T1-weighted	Slightly hyperintense or isointense	0
T1-weighted	Slightly hypointense	1
T1-weighted	Hypointense	2
Site of femoral involvement		
Diaphysis		1
Proximal epiphysis/apophysis		2
Distal epiphysis		3

* Determined in relation to the signal intensity of subcutaneous fat.

Source: Adapted with permission from Maas et al.**^(3)^**.

**Table 2:** L-BMB score, based on MRI signal intensity, together with the patterns of lumbar bone marrow infiltration by Gaucher cells.

Variable	Signal intensity*	L-BMB score
MRI sequence		
T2-weighted	Hyperintense	2
T2-weighted	Slightly hyperintense	1
T2-weighted	Isointense	0
T2-weighted	Slightly hypointense	1
T2-weighted	Hypointense	2
T1-weighted	Slightly hyperintense	0
T1-weighted	Isointense	1
T1-weighted	Slightly hypointense	2
T1-weighted	Hypointense	3
Pattern of lumbar infiltration		
Patchy		1
Diffuse		2
Absence of fat around the basivertebral veins		3

* Determined in relation to the signal intensity of healthy intervertebral discs.

Source: Adapted with permission from Maas et al.**^(3)^**.

### Statistical analysis

Data were analyzed with the IBM SPSS Statistics software package, version 20.0 (IBM Corp., Armonk, NY, USA). We looked for linear relationships among the variables splenic volume, L-BMB score, F-BMB score, BMB score, and quality of life. The Kolmogorov-Smirnov and Shapiro-Wilk normality tests were used in order to determine the distribution of the data. Linear regression analysis was used in order to estimate the slope of the relationship that splenic volume has with the L-BMB, F-BMB, BMB, and SF-36 scores. Values of *p* < 0.05 were considered statistically significant.

## RESULTS

Demographic characteristics and imaging findings are summarized in Table 3. The study population comprised 18 patients (8 men and 10 women), with a mean age of 38.2 years (range, 17-70 years). Bone complications were seen in 9 patients: 7 had bilateral femoral infarcts; and 3 had femoral head avascular necrosis.

**Table 3:** Demographic characteristics and imaging aspects of the patients in the study population (n = 18).

ID	Gender	Age (years)	L-BMB score	F-BMB score	BMB score	Splenic volume (cm^3^)	SF-36 score	Bone complications
1	Male	70	4	6	10	1,616	109.4	Bilateral femoral infarcts
2	Female	41	6	7	13	456	112.4	Bilateral femoral infarcts
3	Female	31	5	4	9	448	114.4	-
4	Male	17	3	3	6	317	120	-
5	Female	20	5	4	9	347	127.8	Femoral head avascular necrosis
6	Male	30	6	4	10	1,231	120.6	Femoral head avascular necrosis
7	Male	23	6	4	10	465	122.8	-
8	Female	65	3	8	11	-	121.5	Bilateral femoral infarcts and femoral head avascular necrosis
9	Male	40	6	8	14	-	118.8	Bilateral femoral infarcts
10	Female	61	5	6	11	323	105.4	Bilateral femoral infarcts
11	Female	32	6	6	12	801	93.8	-
12	Female	24	7	4	11	1,341	112.2	Bilateral femoral infarcts
13	Female	28	7	6	13	2,323	121	Bilateral femoral infarcts
14	Male	27	7	6	13	7,450	108.5	-
15	Male	27	4	2	6	218	114.3	-
16	Female	54	4	4	8	118	100	-
17	Female	50	4	0	4	78	120.5	-
18	Male	18	5	2	7	386	106.5	-

**Figure 1:**
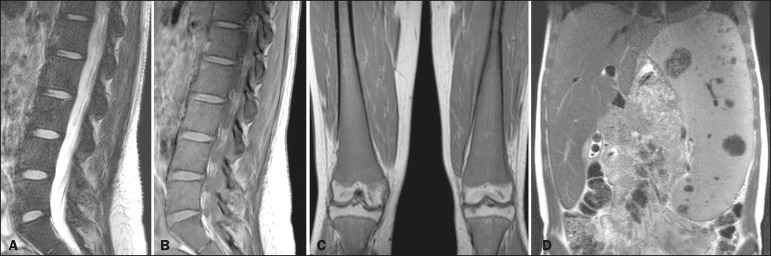
MRI of a 28-year-old woman with GD, showing extensive bone involvement with a high BMB score, together with splenomegaly (splenic volume of 2,323 cm^3)^. Sagittal T2-weighted (A) and T1-weighted (B) images of the lumbar spine, showing lower signal intensity in bone marrow than in the disc and presacral fat. After suppression of the fat surrounding the basivertebral veins, the signal intensity and extent scores were 4 and 3, respectively, resulting in an L-BMB score of 7. C: Coronal T1-weighted image of both femurs, which were scored 3 for signal intensity and 3 for the extent of involvement, infiltration of distal epiphyses being detected, resulting in an F-BMB score of 6. D: Coronal T2-weighted image of the abdomen, demonstrating massive splenomegaly, with multiple splenic infarcts.

**Figure 2:**
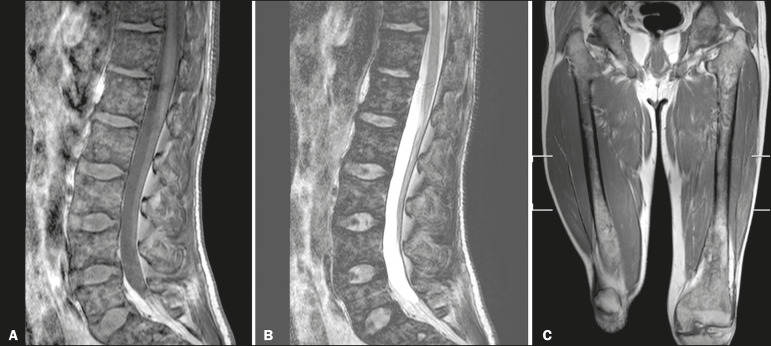
MRI of a 40-year-old man with GD who, despite having extensive bone involvement, did not show poor quality of life, as measured with the SF-36. Sagittal T1-weighted (A) and T2-weighted (B) images of the lumbar spine, showing lower signal intensity in bone marrow than in the disc and presacral fat. The signal intensity score was 5, and the extent score was 1 because the fat surrounding the basivertebral veins was not suppressed, resulting in an L-BMB score of 6. C: Coronal T1-weighted image of both femurs, which were scored 5 for signal intensity and 3 for the extent of involvement, infiltration of epiphyses being detected, resulting in an F-BMB score of 8.

As can be seen in Table 4, the total BMB score ranged from 4 to 14 (mean, 9.83). The L-BMB score ranged from 3 to 7 (mean, 5.17), and the F-BMB score ranged from 0 to 8 (mean, 4.67). Splenic volume ranged from 78 cm^3^ to 7,450 cm^3^ (mean, 1,119.88 cm^3^). Two patients had undergone splenectomy. The SF-36 (quality of life) score ranged from 93.8 to 127.8 (mean, 113.88). Those variables were correlated by means of linear regression models. We found that the splenic volume had linear relationships with the total BMB (Figure 1), L-BMB, and F-BMB scores (*p* < 0.05 for all), the correlations being of equal strength for the L-BMB and F-BMB scores (*r*^2^ = 0.39 and *p* < 0.001 for both). However, the BMB score was a better alternative in this sense, because it presented a lower confidence interval and standard error, which allows greater accuracy in the correlation with the splenic volume (*r*^2^ = 0.40 and *p* < 0.001). The SF-36 score did not show a statistically significant relationship with the splenic volume (*p* = 0.727) or the BMB score (*p* = 0.384) (Figure 2).

**Table 4:** Descriptive statistics for the variables.

Variable	N	Mean	Median	Minimum	Maximum	Standard error
L-BMB score	18	5.17	5	3	7	1.29
F-BMB score	18	4.67	4	0	8	2.14
Total BMB score	18	9.83	10	4	14	2.79
Splenic volume (cm^3^)	16	1,119.88	452	78	7,450	1,799.37
SF-36 score	18	113.88	114.35	93.8	127.8	8.78

## DISCUSSION

GD is an autosomal recessive disease, affecting both genders equally. Some studies have shown a slight predominance in females^(2)^, similar to that observed in our study, in which 55.4% of the patients were women. In addition, the mean age of our patients was 38.2 years (range, 17-70 years), similar to the 34.7 years (range, 1-90 years) reported in the "The Gaucher Registry", a multicenter study conducted in 2000^(25)^.

Bone involvement in type 1 GD usually has a progressive course, leading to disability and dramatic deterioration that may be related to osteoporosis, avascular necrosis, femoral fractures, or spinal fractures^(26,27)^. Most affected patients have bone lesions at diagnosis, and such skeletal involvement, which is often not properly recognized, may be the most debilitating aspect of GD^(27)^. To analyze the involvement of bone marrow and the response to therapy in GD, the BMB score^(3,24)^ has been shown to correlate well with other measures of bone marrow infiltration, such as Dixon quantitative chemical shift imaging, the BMB score having the advantage of being more widely available because it can be determined from standard MRI sequences without the need for proprietary software and special sequences^(1,27-29)^.

In the present study, we detected bone complications in 9 of the 18 patients evaluated, the main ones being femoral infarction and avascular necrosis of the femoral head. The evaluation and monitoring of bone involvement is an important element in the clinical management of GD, allowing treatment to be optimized before the complications become irreversible^(11)^. The risk of infarction and avascular necrosis has been shown to be lower in patients who begin ERT within two years after diagnosis than in those who experience delays of more than two years between diagnosis and treatment^(19,20)^. That implies that early diagnosis has prognostic value in GD.

In type 1 GD, bone involvement may not reflect the involvement of other organs; severe skeletal disease may occur in patients with mild or no visceral and hematological involvement^(18-20)^. Nevertheless, our study demonstrated a good correlation between the BMB scores and splenic volume, the total BMB score showing a stronger correlation than that observed for the segmented (L-BMB and F-BMB) scores. That suggests that there is a relationship between the presence of aggressive bone disease (characterized by high BMB scores) and visceral involvement (characterized by increased splenic volume) in GD. However, we found no significant correlation between the BMB score and the SF-36 score. That is probably due to the insidious and silent nature of type 1 GD, which causes indolent and progressive bone changes, with a tendency to affect patient quality of life only later, especially when irreversible bone damage has already occurred. Therefore, early diagnosis of type 1 GD, which often occurs in the asymptomatic phase of the disease, and quantification of the extent of bone disease, increase the likelihood of appropriate treatment and prevention of long-term complications, with the subsequent benefit of improving the quality of life of the patients.

In conclusion, our study demonstrated a linear relationship between the BMB score and splenic volume, corresponding to more extensive bone disease in individuals with massive splenomegaly. No correlation was found between the extent of bone disease and quality of life, illustrating the insidious and silent progression of GD. Therefore, we emphasize the importance of early diagnosis and monitoring of bone changes in patients with GD, even in those who are asymptomatic, in order to provide appropriate treatment and prevent bone complications, thus improving the prognosis and quality of life of these patients.
